# DECIMER—hand-drawn molecule images dataset

**DOI:** 10.1186/s13321-022-00620-9

**Published:** 2022-06-09

**Authors:** Henning Otto Brinkhaus, Achim Zielesny, Christoph Steinbeck, Kohulan Rajan

**Affiliations:** 1 Institute for Inorganic and Analytical Chemistry, Friedrich-Schiller-University Jena, Lessingstr. 8, 07743 Jena, Germany; 2 Institute for Bioinformatics and Chemoinformatics, Westphalian University of Applied Sciences, August-Schmidt-Ring 10, 45665 Recklinghausen, Germany

## Abstract

The translation of images of chemical structures into machine-readable representations of the depicted molecules is known as optical chemical structure recognition (OCSR). There has been a lot of progress over the last three decades in this field, but the development of systems for the recognition of complex hand-drawn structure depictions is still at the beginning. Currently, there is no data for the systematic evaluation of OCSR methods on hand-drawn structures available. Here we present *DECIMER — Hand-drawn molecule images*, a standardised, openly available benchmark dataset of 5088 hand-drawn depictions of diversely picked chemical structures. Every structure depiction in the dataset is mapped to a machine-readable representation of the underlying molecule. The dataset is openly available and published under the CC-BY 4.0 licence which applies very few limitations. We hope that it will contribute to the further development of the field.

**Graphical Abstract:**

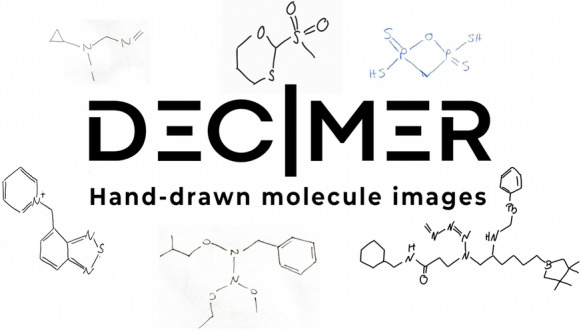

## Objective

Most chemical information is published in text and images in the primary scientific literature. The automated conversion of these unstructured, human-readable data formats into structured, machine-readable representations is essential to make the information available in publicly accessible databases. The reliable extraction of information from the depictions of the chemical structures is an ongoing challenge that still has not been fully solved yet. Chemical structure depictions are converted into computer-readable representations using optical chemical structure recognition (OCSR) systems [1].

The field of OCSR has developed significantly over the last 30 years. Most OCSR tools follow a hard-coded set of rules to assemble the underlying molecule based on the elements in the vectorised image [2–11]. By 2020 several deep learning-based solutions are available [12–18].

In order to evaluate the performance of the available OCSR tools, realistic benchmark datasets are necessary. At present, there are four real-world datasets available [1, 9, 19] that contain chemical structure depictions that were collected and curated from publications and patents. The evaluation of the performance on realistic data is crucial to demonstrate whether the tools are robust enough to be used in an automated chemical literature mining process.

The resolution of hand-drawn chemical structures is a more challenging task than the resolution of automatically generated depictions. In addition to the varying depiction features which are present anyway, the individual, unique way of drawing the structure adds an increased level of complexity. In 2021, the deep learning-based OCSR tool ChemPix [15] demonstrated its capability to interpret simple hand-drawn hydrocarbon structures with high accuracy. There also are a few closed-source methods and commercial systems available that claim to be capable of resolving hand-drawn chemical structures [20–22]. The authors of the deep-learning-based OCSR tool img2mol demonstrated the capability of their tool to recognise some hand-drawn chemical structures that they had picked themselves and noted the lack of a standardised benchmark set [14].

With the development of more OCSR tools that focus on the resolution of hand-drawn chemical structure depictions, there is a need for a standardised dataset to evaluate their performance. Here we present *DECIMER — Hand-drawn molecule images*, a set of 5088 hand-drawn chemical structures depictions. Every image is mapped to a machine-readable representation of the underlying molecule. The diversely picked molecules represent a wide variety of small molecules. The dataset was created to facilitate the ongoing development in the field of OCSR and is openly accessible.

## Data description

**Fig. 1:**
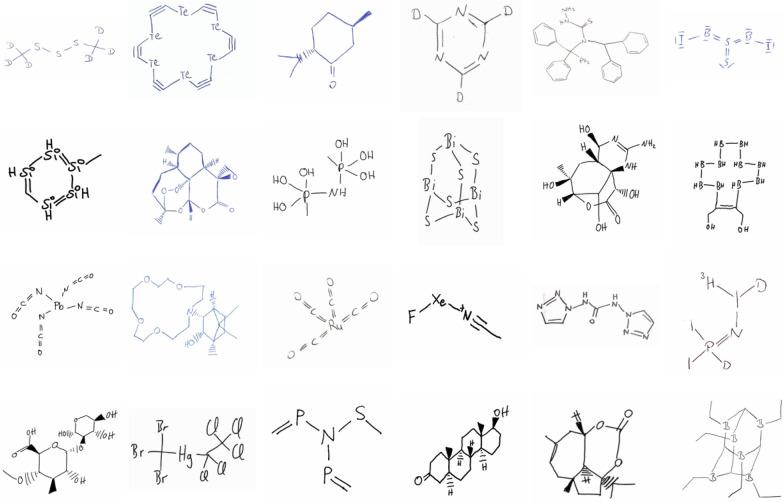
Examples of hand-drawn chemical structure depictions from the dataset

The dataset consists of 5088 PNG images of unique hand-drawn chemical structure depictions (Fig. 1) which are mapped to their corresponding SMILES [23] string as well as an SD file. The structures have been drawn by 24 volunteers from the Westphalian University of Applied Sciences, Campus Recklinghausen, Germany, who have graciously offered to use their free time to contribute to the generation of this dataset.

The molecules have been picked from all structures in PubChem [24] using RDKit’s implementation of the MaxMin algorithm [25] based on Morgan fingerprints [26] to ensure a diverse coverage of the chemical space. The only filtering rule that has been applied is a molecular weight maximum of 1500 Da. As a consequence, features like stereochemical information, charged groups as well as different types of isotopes are present in the dataset.

**Fig. 2:**
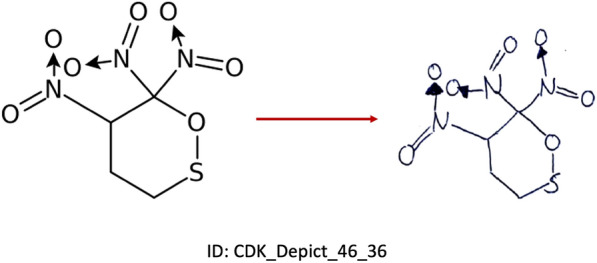
A chemical structure depiction generated by CDK, sketched on a sheet of paper and scanned as an image file

**Fig. 3:**
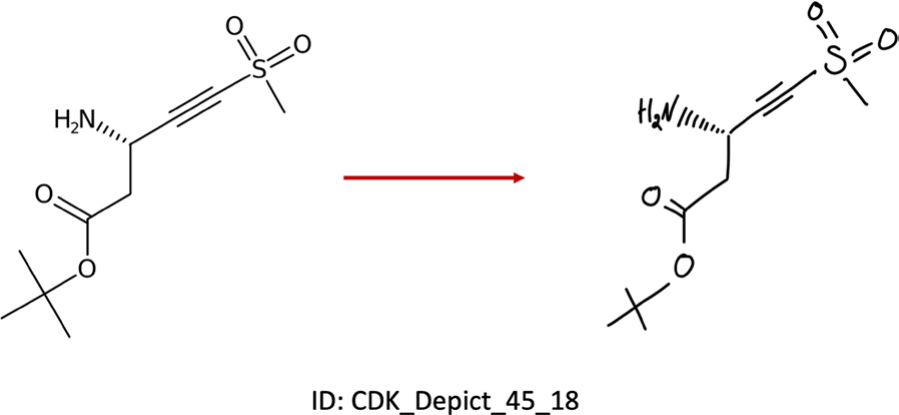
A chemical structure depiction generated by CDK, sketched on a tablet and saved as an image file

There are two categories of images:

- Drawn on a piece of white paper and scanned (Fig. 2)
- Drawn using a mobile device or tablet and directly saved as an image (Fig. 3).

## Curation

In total, 6000 diverse molecules were selected from PubChem using RDKit’s implementation of the MaxMin algorithm based on Morgan fingerprints. Subsequently, CDK Depict [27], a structure depiction generator based on the Chemistry Development Kit (CDK) [28], was used to create production-quality 2D images in batches. Each batch of images was then converted into PDF files and they were distributed among the volunteers. Using the chemical structure depictions generated by CDK as a visual template, each volunteer drew the structures on a piece of paper using a black or blue pen or on their tablet using an input device.

Each volunteer sent back the scanned images or the images generated using their device after completing a batch. The curators reviewed the drawings, manually confirmed the correctness of the molecules, cropped the scanned images and stored them in separate image files. As part of the curation, structures that weren't correct due to human error were discarded. A total of 568 images out of 6000 were rejected due to issues with the depicted structure. Another 344 structures were not returned by the volunteers. This resulted in the final dataset of 5088 images in total.

An identifier was assigned to each image, and the same identifier was used to label the SD file which was generated using the CDK. Additionally, the dataset contains a file containing a table of the identifiers and corresponding SMILES representations.

## FAIR-ification

The following steps were taken in order to make the dataset findable, accessible, interoperable and reusable (FAIR) [29]. The dataset was deposited in a publicly accessible data repository, in this case, Zenodo. This ensures that the dataset is easily findable. Furthermore, Zenodo provides a digital object identifier (DOI) that can be used to locate the dataset and it can also easily be integrated into Github as well. With Zenodo being an open, public repository, the dataset can be accessed from any part of the globe. To make it as interoperable as possible, the generated images use PNG as the final image format, which can be used across a variety of operating systems. Additionally, SMILES and SDF are representations of chemical structures which can be read by every cheminformatics toolkit. The dataset has been published under the CC-BY 4.0 licence. This licence includes that every user can redistribute or change the data as much as they want as long as they refer to the original authors when publishing results based on it. It is possible to use the data for non-commercial or commercial purposes without further obligations.

## Limitation

No restrictions or limitations apply to using and reusing the dataset. Everyone can use this dataset as a standard benchmark set for the evaluation of the performance of their OCSR tools. The dataset includes a wide range of chemical structures and represents a much larger chemical space. The structures were drawn by various individuals to ensure the diversity of drawing styles. The main limitation is caused by the molecular weight filter (< 1500Da) as it excludes certain molecules like big macrocycles, proteins or artificial polymers. Additionally, Markush structures are not represented.

Due to the limited number of images in this dataset, we do not recommend attempting to train a deep learning model using this dataset. We highly recommended using it exclusively for benchmarking instead of fitting the tools to the dataset.

## Data Availability

The dataset is openly available at ZENODO: https://doi.org/10.5281/zenodo.6456306.

## Abbreviations

CDK: Chemistry development kit
CC: Creative commons
DOI: Digital object identifier
FAIR: Findable, accessible, interoperable, and reusable
OCSR: Optical chemical structure recognition
PDF: Portable document format
PNG: Portable network graphics
SDF: Structural data file
SDG: Structure diagram generator
SMILES: Simplified molecular-input line-entry system
